# Doxycycline inhibits breast cancer EMT and metastasis through PAR-1/NF-κB/miR-17/E-cadherin pathway

**DOI:** 10.18632/oncotarget.20418

**Published:** 2017-08-24

**Authors:** Weilong Zhong, Shuang Chen, Yuan Qin, Heng Zhang, Hongzhi Wang, Jing Meng, Longcong Huai, Qiang Zhang, Tingting Yin, Yueyang Lei, Jingxia Han, Lingfei He, Bo Sun, Huijuan Liu, Yanrong Liu, Honggang Zhou, Tao Sun, Cheng Yang

**Affiliations:** ^1^ State Key Laboratory of Medicinal Chemical Biology and College of Pharmacy, Nankai University, Tianjin, 300000, China; ^2^ Tianjin Key Laboratory of Molecular Drug Research, Tianjin International Joint Academy of Biomedicine, Tianjin, 300000, China

**Keywords:** doxycycline, miR-17, EMT, GPCR, NF-κB

## Abstract

Doxycycline displays high efficiency for cancer therapy. However, the molecular mechanism is poorly understood. In our previous study, doxycycline was found to suppress tumor progression by directly targeting proteinase-activated receptor 1 (PAR1). In this study, microRNAs were found to be involved in PAR1-mediated anti-tumor effects of doxycycline. Among these miRNAs, miR-17 was found to promote breast cancer cell metastasis both *in vivo* and *in vitro*. Moreover, miR-17 could reverse partial doxycycline inhibition effects on breast cancer. Employing luciferase and chromatin immunoprecipitation assays, nuclear factor-kappaB (NF-κB) was found to bind miR-17 promoters. Furthermore, E-cadherin was identified as the target gene of miR-17. These results showed that miR-17 can resist the inhibitory effects of doxycycline on breast cancer epithelial–mesenchymal transformation (EMT) by targeting E-cadherin.

## INTRODUCTION

Breast cancer is a prevalent malignancy and the leading cause of cancer death among women worldwide [1]. Various therapies are used for breast cancer treatments; these interventions include surgery, chemotherapy and targeted therapy [2–6]. In addition to its antimicrobial activity, doxycycline was reported to display strong efficacy for cancer therapy as an antimicrobial drug [7, 8]. Researches studied important roles of doxycycline in breast cancer metastasis and tumor growth [9–11].

Successful breast cancer treatment relies on better understanding of molecular mechanisms involved in cancer progression. Protease-activated receptor 1 (PAR1) is a G protein-coupled receptor involved in metastatic and invasive cancer processes [12–16]. Our previous studies indicated that in PAR1-dependent manner, doxycycline possesses higher inhibition ability in lung cancer and breast cancer [8, 11].

miRNAs comprise a class of small noncoding RNAs modulating gene expression at the post-transcriptional level by repressing translation and promoting destabilization of target mRNAs [17–21]. In the past ten years, thousands of miRNAs were identified [22]. Importance in tumor genesis and progression of miRNAs attracted the attention of scientists and considered for funding by the government. In the present studies, doxycycline repressed miR-17 expression [23–26]. Ectopic expression of miR-17 partially reverses inhibitory effect of doxycycline on breast cancer invasion and tumor genesis. Nuclear factor NF-κB mediates regulation of miR-17 by doxycycline [13, 27–30]. Luciferase activity assay indicated that miR-17 directly targets E-cadherin and represses its expression. Therefore, doxycycline inhibits breast cancer invasion and tumor genesis through PAR1/NF-κB/miR-17/E-cadherin pathway. Such results provide deeper understanding of doxycycline and importance of miRNA in cancer therapy.

## RESULTS

### PAR1 is highly expressed in breast cancer

**Figure 1:**
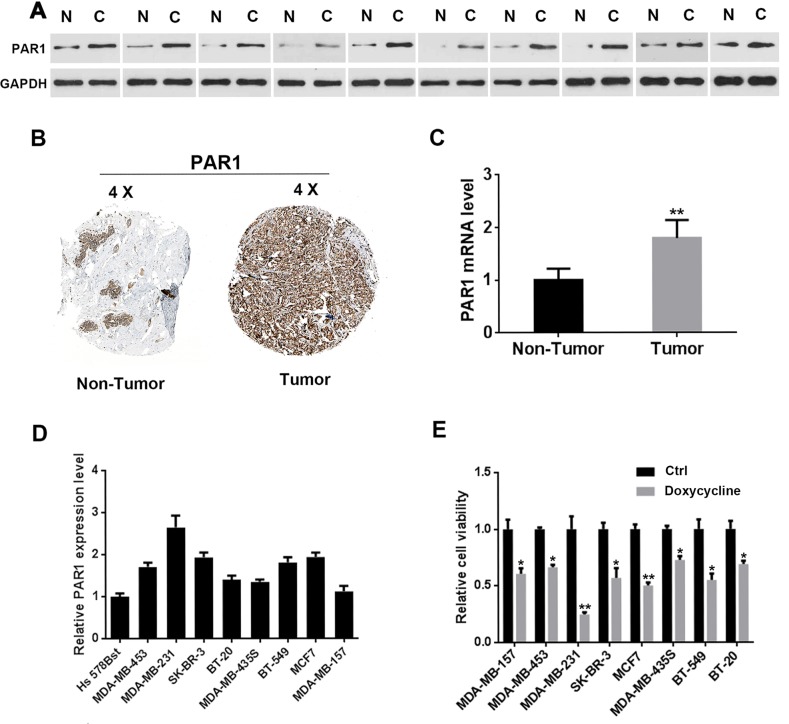
PAR1 is upregulated in human breast cancer **(A)** Western blot analysis of tumor tissues and adjacent tissues of ten pairs of clinical specimens. PAR1 was highly expressed in tumor tissues. **(B)** PAR1 expression levels in normal breast tissues and breast cancer specimens. Images were taken from the Human Protein Atlas (http://www.proteinatlas.org) online database. **(C)** PAR1 mRNA expression levels in normal vs tumor of the breast (n = 10). **(D)** PAR1 was highly expressed in breast cancer cells. PAR1 expressed the highest level in MDA-MB-231 cells. **(E)** Breast cancer cells with higher PAR1 expression levels were more sensitive to doxycycline compared with other cells. Results were obtained from three independent experiments, each performed in triplicate, and the error bars represent the standard deviation (^*^P < 0.05, ^**^P < 0.01). Data is represented as mean ± SEM.

To verify PAR1 expression in breast cancer, 10 pairs of clinical specimens and cell lines were used. Western blot analysis showed that PAR1 protein levels were higher in tumor tissues than those in adjacent tissues (Figure 1A). PAR1 expression in normal breast tissues and breast cancer specimens was investigated and the results showed that the expression levels of PAR1 in breast cancer tissues were higher than those in non-tumor tissues. Images were taken from the Human Protein Atlas (http://www.proteinatlas.org) online database (Figure 1B) [31, 32]. PAR1 mRNA expression levels in normal vs tumor tissues of the breast were detected and results were shown in Figure 1C. Quantitative reverse transcription Polymerase Chain Reaction (qRT-PCR) was applied to test PAR1 expression in breast cancer cell lines. Results showed that PAR1 was expressed the highest in MDA-MB-231 cells (Figure 1D). These high PAR1 expression cell lines were more sensitive to doxycycline compared with low PAR1 expression cells (Figure 1E).

### Doxycycline inhibits breast cancer cell epithelial–mesenchymal transition (EMT) through PAR1/NF-κB signaling pathway

**Figure 2:**
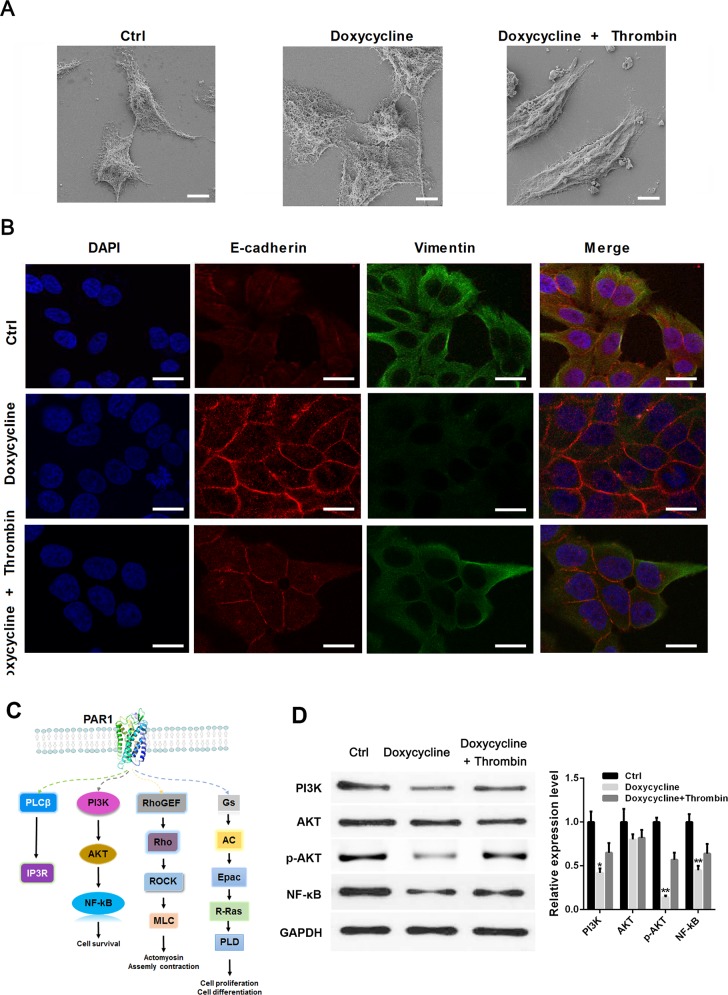
Doxycycline inhibits EMT by increasing the expression of E-cadherin and decreasing the expression of Vimentin through PAR1/PI3K/AKT/NF-κB signaling pathway **(A)** Doxycycline inhibits cell pseudopodia and changes of cell morphology to epithelial phenotype. **(B)** Doxycycline increases E-cadherin expression and decreases Vimentin expression, whereas thrombin partly counteracts this inhibitory effect in MCF-7 cells. **(C)** Proteomics analysis of doxycycline-treated and control groups. Possible pathways inhibited by doxycycline. **(D)** Western blot analysis confirmed the changes of PAR1/NF-κB pathway after doxycycline and thrombin treatment. Results were obtained from three independent experiments, with each experiment performed in triplicate.

To verify whether doxycycline plays its inhibitory function in breast cancer cells through PAR1, thrombin was used to activate PAR1 in MCF-7 cells, E-Cadherin (a classical and most well-studied member of the cadherin superfamily, acting as a canonical epithelial marker) and Vimentin were detected with immunofluorescence assay [33]. SEM and immunofluorescence results indicated that doxycycline could promote cell junction and inhibit EMT, whereas thrombin can counteract this inhibitory effect in MCF-7 cells (Figures 2A and 2B). Figure 2A showed that doxycycline treated cells formed a reduction in lamellipodia and filopodia compared with the control group. In doxycycline and thrombin treated group, the cell morphology was similar as control group, cells change from epithelial phenotype to mesenchymal phenotype. To analyze the mechanism of doxycycline during EMT progression in breast cancer, downstream signaling pathways of PAR1 were analyzed (Figure 2C). Western blot analysis revealed that PAR1/AKT/NF-κB signal pathway changes after doxycycline and thrombin treatments.

### NF-κB directly promotes expression of miR-17

**Figure 3:**
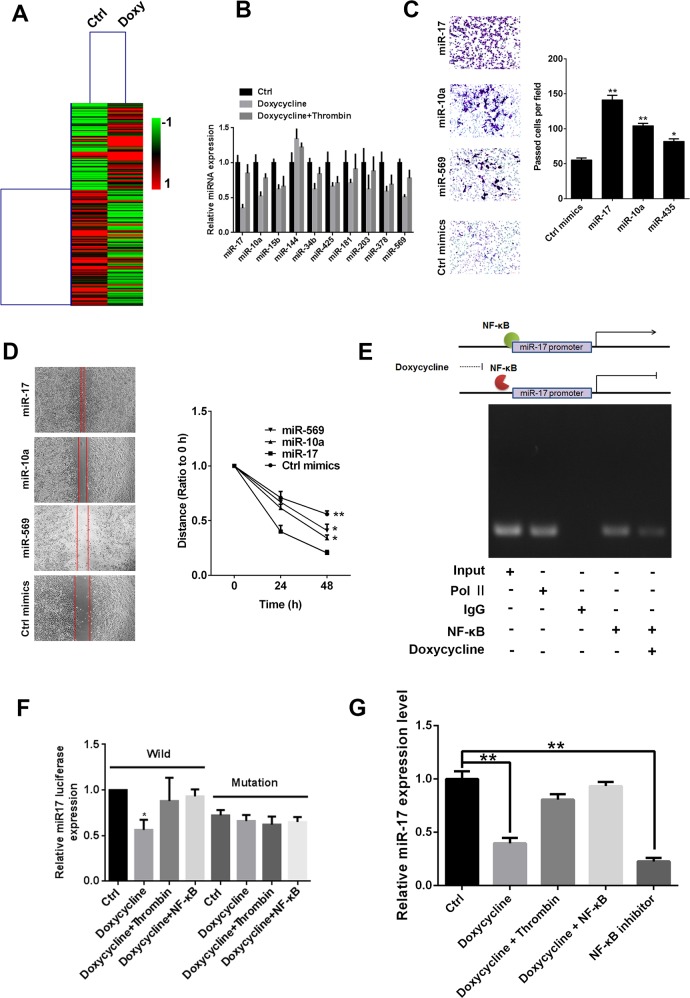
miR-17 promotes tumor progression, and NF-κB directly promotes expression of miR-17 **(A)** Cluster analysis of microarray expression profile. **(B)** miRNA-specific qRT-PCR was conducted to examine expression of candidate mi-RNAs. **(C)** Transwell assay of miR-17, miR-10a and miR-569; miR-17 significantly increased invasiveness of breast cancer cells. **(D)** Scratch assay of miR-17, miR-10a and miR-569; miR-17 significantly increased migration ability of breast cancer cells. **(E)** Effect of NF-κB on miR-17 promoter. Results showed that NF-κB directly binds to miR-17 promoter and upregulates its transcription **(F)** luciferase reporter assay evaluate effects of doxycycline and NF-κB on miR-17 promoter, results shown that doxycycline had inhibit effects on Wild, while loss inhibit activity on mutation miR-17. NF-κB binding miR-17 promoter and counteracts doxycycline inhibitory effect. **(G)** Relative miR-17 expression levels in different treatment groups. Doxycycline inhibits miR-17 expression. Results were obtained from three independent experiments, with each experiment performed in triplicate; error bars represent standard deviation (^*^P < 0.05, ^**^P < 0.01). Data are represented as mean ± SEM.

Using microarray expression profiles, we obtained differentially expressed microRNAs in MCF-7 cells after treatment with doxycycline alone or doxycycline with thrombin (Figure 3A). Then, miRNA-specific qRT-PCR was performed to validate candidate miRNAs. Results revealed three miRNAs that are more sensitive to doxycycline, miR-17, miR-10a, and miR-569 (Figure 3B). Transwell and wound healing assay results showed that all three miRNAs can promote cell migration and invasion; miR-17 presented the most remarkable effect (Figures 3C and 3D). To further explore whether this doxycycline effect is mediated by miR-17, a luciferase reporter assay was performed to evaluate effects of NF-κB on miR-17 promoter (Figures 3E and 3F). Results showed that NF-κB directly binds to promoter of miR-17 and unregulated its transcription (Figure 3G).

### miR-17 counteracts partial doxycycline inhibitory effect on breast cancer cells

**Figure 4:**
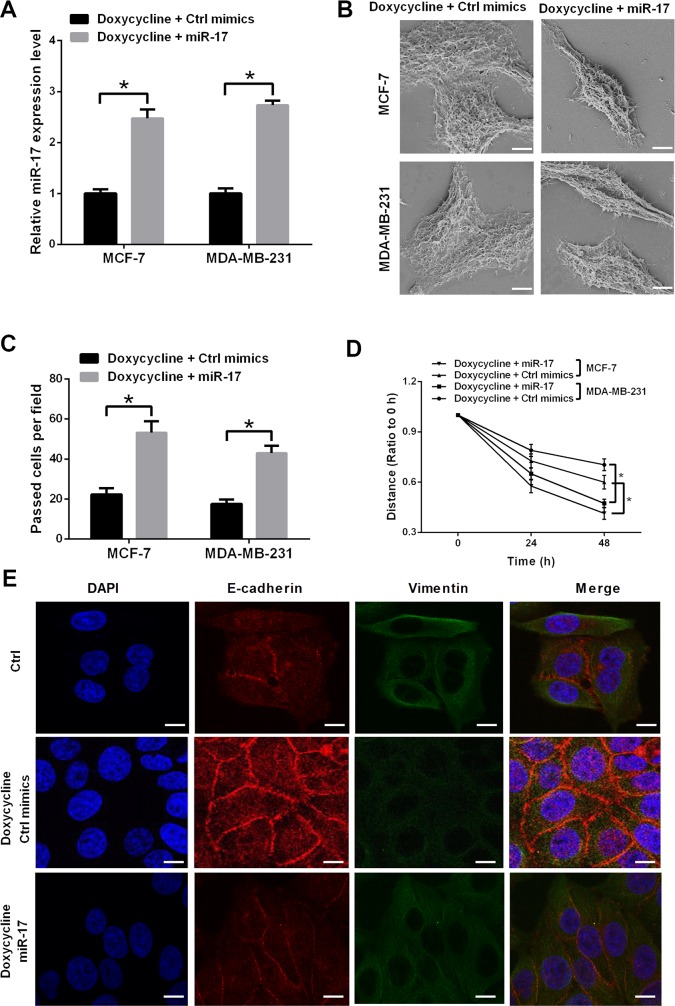
miR-17 induces EMT and counteracts partial inhibitory effect of doxycycline on MCF-7 and MDA-MB-231 cells **(A)** Relative miR-17 expression levels in doxycycline and doxycycline/miR-17 groups. **(B)** Cell morphology of MCF-7 and MDA-MB-231 cells. Cell morphology changes to epithelial phenotype; mesenchyme is notable in doxycycline/miR-17 cells. **(C)** Transwell assay of doxycycline and doxycycline/miR-17 groups. miR-17 increases invasiveness of MCF-7 and MDA-MB-231 cells. **(D)** Scratch assay of doxycycline and doxycycline/miR-17 groups. miR-17 increases migration ability of MCF-7 and MDA-MB-231 cells. **(E)** Immunofluorescence assay of doxycycline and doxycycline/miR-17 groups. In doxycycline-pretreated cells, miR-17 can upregulate Vimentin and downregulate E-cadherin expression levels. Results were obtained from three independent experiments, with each experiment performed in triplicate; error bars represent standard deviation (^*^P < 0.05, ^**^P < 0.01). Data are represented as mean ± SEM.

To verify whether doxycycline suppressor function in breast cancer happens through miR-17, a rescue experiment was performed. MCF-7 and MDA-MB-231 cells were treated with doxycycline alone or doxycycline with miRNA mimics. In miR-17 transfected group, the relative miR-17 expression level was higher than that in control group, both in MCF-7 and MDA-MB-231 cells, this result indicated that miR-17 expression was up-regulated after miR-17 mimic transfection (Figure 4A). In SEM (scanning electron microscopy) analysis, miR-17 up-regulation drove cell morphology to change from epithelial phenotype to mesenchymal phenotype and then attenuated cell junction in breast cancer cells (Figure 4B). Wound healing and transwell assays indicated that miR-17 upregulation can restore doxycycline-inhibited cell invasion and migration (Figures 4C and 4D). In doxycycline-pretreated cells, miR-17 implication can also upregulate Vimentin and downregulate E-cadherin expression levels. Therefore, miR-17 can restore doxycycline-inhibited EMT in breast cancer cells (Figure 4E).

### miR-17 directly targets E-cadherin

**Figure 5:**
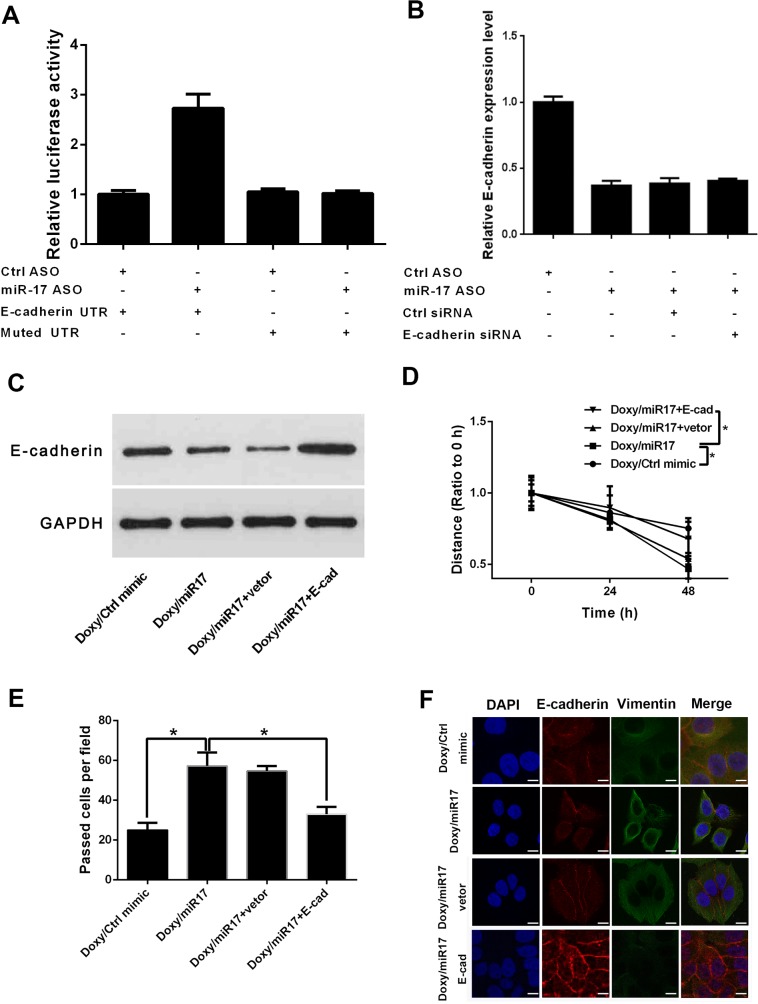
miR-17 downregulates expression of E-cadherin and promotes EMT of breast cancer cells **(A)** Luciferase reporter assay of E-cadherin. Knock-down of miR-17 reduced binding of miRNA to E-cadherin UTR and increased luciferase activity **(B)** Relative E-cadherin expression levels in miR-17- and E-cadherin siRNA-treated groups. **(C)** Western blot analysis of E-cadherin in miR-17- and E-cadherin-siRNA-treated groups. **(D)** Wound healing analysis of E-cadherin in miR-17- and E-cadherin-siRNA-treated groups. Results suggested that ectopic E-cadherin expression can reverse miR-17-induced repression of E-cadherin expression. miR-17 increases cell migration abilities, and ectopic E-cadherin expression counteracts the effects of miR-17. **(E)** Transwell assay confirmed that ectopic E-cadherin expression counteracts effects of miR-17 on breast cancer cells. **(F)** Immunofluorescence assay of miR-17- and E-cadherin-siRNA-treated groups. Results showed that E-cadherin reverses the effect of miR-17 on breast cancer cell EMT. Results were obtained from three independent experiments, with each experiment performed in triplicate; error bars represent standard deviation (^*^P < 0.05, ^**^P < 0.01). Data are represented as mean ± SEM.

To further test the exact mechanism of miR-17 in doxycycline regulation of breast cancer, downstream targets of miR-17 were predicted and verified. In luciferase reporter assay, miR-17 knock-down reduced miRNA binding to E-cadherin untranslated region (UTR) and increased luciferase activity (Figure 5A). qRT-PCR and Western blot results suggested that ectopic E-cadherin expression can reverse miR-17-induced repression of E-cadherin expression (Figures 5B and 5C). Wound healing and transwell assays confirmed that ectopic E-cadherin expression counteracts the effect of miR-17 on breast cancer cell invasion and migration (Figures 5D and 5E). Immunofluorescence results revealed that E-cadherin reversed the effect of miR-17 on breast cancer cell EMT (Figure 5F).

### miR-17 reverses inhibition effect of doxycycline on tumor progression *in vivo*

**Figure 6:**
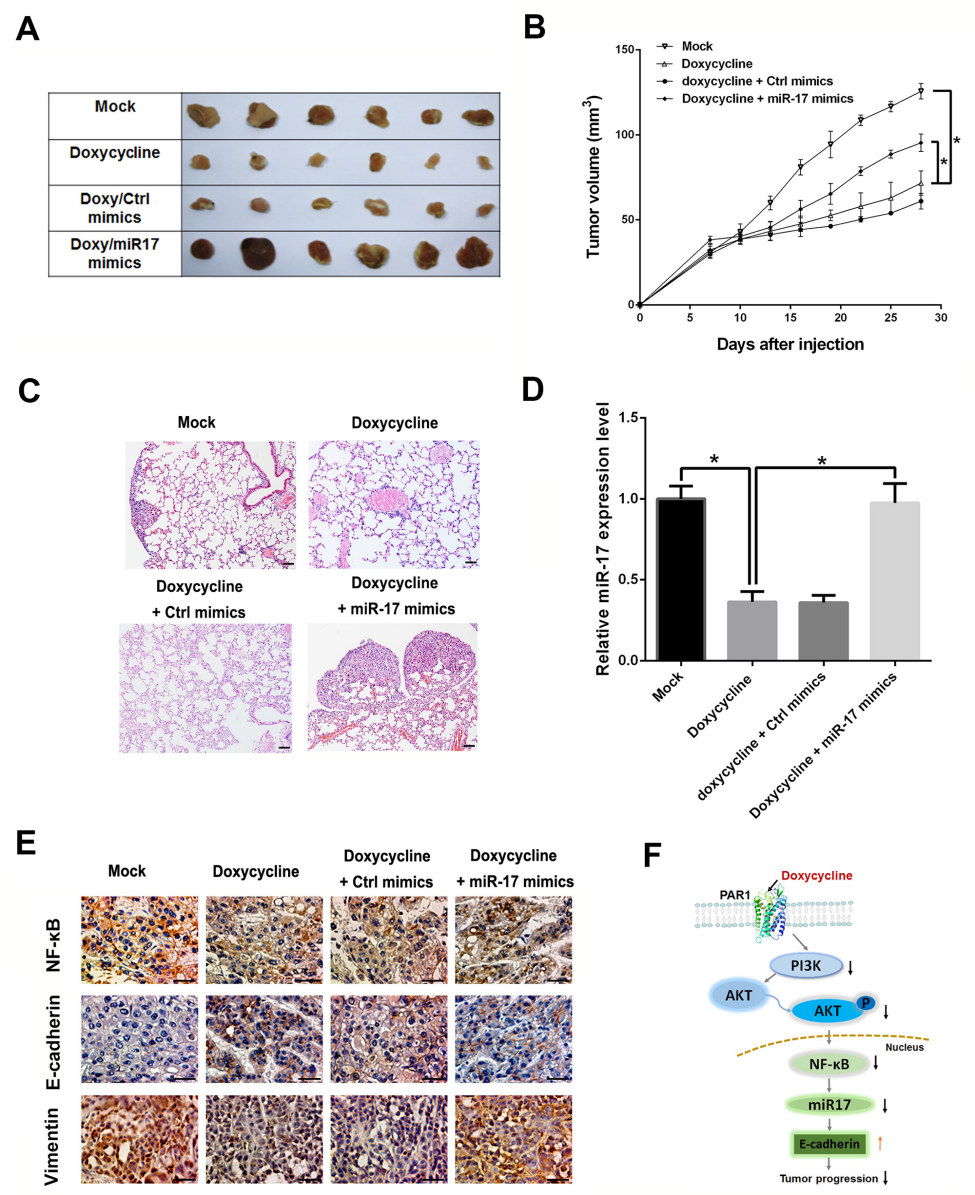
Doxycycline inhibits tumor progression, whereas miR-17 reverses inhibitory effect of doxycycline **(A)** Xenograft assays in doxycycline and doxycycline/miR-17 groups. **(B)** Tumor volumes in mice treated with doxycycline and doxycycline/miR-17 groups. Tumor volumes of the doxycycline-treated group are smaller than those of control group, whereas tumor volumes rebounded in mice treated with miR-17 and doxycycline **(C)**. Metastasis analysis of doxycycline and doxycycline/miR-17 groups. miR-17 restoration can promote lung metastasis of breast cancer. **(D)** Relative miR-17 expression levels in qRT-PCR. Results indicated that miR-17 expressions were reduced after doxycycline treatment. **(E)** IHC analysis of E-cadherin, Vimentin and NF-κB. Compared with the mock group, in Doxycycline treatment groups, NF-κB and Vimentin were stainted lighter, whereas E-Cadherin was stained darker. In the Doxycycline/miR-17 treatment group, however, NF-κB and Vimentin were stained slightly lighter than those in mock group but darker than those in Doxycycline treatment groups while E-Cadherin was stained darker than that in mock group and similar with or slightly lighter than those in Doxycycline treatment groups. IHC results showed that miR-17 can promote EMT progression, upregulate Vimentin, and repress E-cadherin expression. **(F)** Mechanism of doxycycline inhibits tumor progression. Results show that doxycycline inhibits tumor progression through PAR-1/NF-κB/miR-17/E-cadherin pathway. The HE images were 100×, IHC images were 400×. Results were obtained from three independent experiments, with each experiment performed in triplicate; error bars represent standard deviation (^*^P < 0.05, ^**^P < 0.01). Data are represented as mean ± SEM.

To investigate the role of miR-17 *in vivo*, MCF-7 cells were inoculated subcutaneously in mouse axillae. Mice were sacrificed after treatment with doxycycline alone or doxycycline with miR-17 for 28 days. In mice treated with doxycycline alone, tumor volumes were smaller than those in control group, whereas tumor volumes rebounded in mice treated with miR-17 and doxycycline (Figures 6A and 6B). Doxycycline inhibit lung metastasis, while miR-17 restoration can promote lung metastasis of breast cancer (Figure 6C). qRT-PCR and IHC were performed to analyze expressions of miR-17 and EMT markers in solid tumors. Results indicated that miR-17 expressions were reduced and E-Cadherin expressions were up-regulated after doxycycline treatment (Figure 6D and 6E). Immunohistochemical staining for E-cadherin results shown that the E-cadherin were highly expressive in doxycycline treated group compared with the untreated group. miR-17 can promote EMT progression, up-regulate Vimentin and reduce E-cadherin expressions (Figure 6E). These results imply that miR-17 restoration can promote breast cancer cell metastasis through EMT regulation. Figure 6F displays the mechanism of doxycycline inhibits tumor progression. Doxycycline inhibits PAR1 and downstream NF-κB/miR-17/E-cadherin pathway to suppress tumor progression.

## DISCUSSION

As a member of the tetracycline family, doxycycline affects angiogenesis and metastasis in tumor progression [34, 35]. Previous study implied that doxycycline could induce EMT by regulating TMPRSS2 in LNCaP cells. While other researches demonstrated that doxycycline could inhibit the progression of cancer stem cell phenotype and EMT through FOXM1 and IκB/NF-κB signal pathway. To explore the molecular mechanism of anti-tumor effects of doxycycline, PAR1 was identified as a direct target in our previous study. PAR1 belongs to G protein-coupled receptors, which are involved in metastatic and invasive cancer processes [36]. Critical effects of PAR1 in tumor occur through multiple signal pathways. Malik supported that NF-κB activation is induced by PAR1 [13]. NF-κB is a well-known transcriptional factor that is important in tumor progression and regulates various gene expressions, including those of protein-coding genes and non-coding RNAs [13, 37]. We observed that doxycycline inhibits NF-κB via PAR1.

miR-17 belongs to NF-κB-regulated miRNAs driving key physiological responses during tumor genesis and proliferation. In breast cancer, miR-17 is overexpressed and is associated with cell metastasis and proliferation [24]. In the present study, miR-17 was highly expressed in breast cancer cells and resisted doxycycline inhibition of cell migration and invasion. miR-17 also directly targets E-cadherin and represses its expression at post-transcription level [38]. E-cadherin dysregulation is correlated with EMT progression. Down-regulation of E-cadherin contributes to cancer progression by increasing proliferation and invasion. In the present work, miR-17 directly decreased E-cadherin and contributed to migration and invasion of breast cancer cells.

In summary, the current work indicated that inhibitory effects of doxycycline on breast cancer cell occur through a miR-17-dependent pathway. Both *in vitro* and *in vivo* experiments showed that binding of doxycycline to PAR1 leads to NF-κB inactivation, which subsequently down-regulates miRNA and up-regulates E-cadherin. Up-regulation of E-cadherin inhibits EMT progression and decreases invasion ability of breast cancer cells. These results support a model, in which repression of PAR1 by doxycycline upregulates E-cadherin expression by inactivating NF-κB and miR-17, thereby inhibiting the invasion of breast cancer cells. The present study provides a deeper understanding of the molecular mechanism of doxycycline in tumor regulation and may assist cancer therapies using tetracycline.

## MATERIALS AND METHODS

### Cell culture

Human breast cell lines MCF-7 and MDA-MB231 were purchased from KeyGen Biotech (Nanjing, China). These cells were maintained in Roswell Park Memorial Institute (RPMI)-1640 medium (Hyclone, USA) supplemented with 10% fetal bovine serum (FBS) (Hyclone, USA) at 37°C in humidified atmosphere containing 5% CO_2,_ trypsinized and passaged every two days.

### Immunofluorescence staining

Transfected cells were fixed with 4% formaldehyde in phosphate-buffered saline (PBS) for 5 min. After permeabilization with 0.2% Triton X-100 and blocked with 3% bovine serum albumin, the cells were incubated overnight with primary antibodies at 4°C. FITC(fluorescein isothiocyanate) or TRITC(tetraethyl rhodamine isothio cyanate)-labeled secondary antibodies (Earthox LLC., USA) was then incubated for 1 h at room temperature. Each step was followed by two 5 min PBS washings. Finally, cells were stained with DAPI (4′, 6-diamidino-2-phenylindole) (Sigma, USA), mounted and viewed using the laser scanning confocal microscope A1 (Nikon, Japan).

### Scanning electron microscopy (SEM)

Cells were grown on climbing films and treated with doxycycline (1 μM). The same volume of ddH2O containing the same percent of DMSO was added in the control group. After 24 h, cells were fixed and dehydrated in acetone/isoamyl acetate (1:1) and dried with a gradient concentration of acetonitrile. After coating with gold, cells were photographed using a scanning electron microscope (LEO 1530 VP, Germany).

### Matrigel invasion assay

A total of 5 × 10^4^ cells were seeded in top-chamber inserts coated with matrigel (BD Biosciences) with RPMI-1640 containing 2% FBS. The bottom chamber was filled with 500μL Dulbecco's modified Eagle's medium (DMEM)containing 10% FBS. Doxycycline (1 μM) were added to the upper chamber. After 24 h of incubation, inserts were washed three times with 1×phosphate-buffered saline (PBS) gently and the cells transferred through the filter membrane of the chambers were fixed in 4% paraformaldehyde (precooled at 4°C) and stained with 0.1% crystal violet. The passed cells were counted under a microscope (Nikon, Japan).

### Wound healing assay

Cells were grown in 24-well culture plates at a density of 2×10^5^ cells/well. 24 h later, a wound was made in the center of the well. After treatment with doxycycline (1μM) and mimics, wound images were photographed after 24 and 48 h using a light microscope (Nikon, Japan).

### Murine Xenograft Model

Six-week-old female BALB/c-null mice were used to test the effects of doxycycline and miR-17 on breast cancer. Animal experiments were conducted in accordance with National Institutes of Health Animal Use Guidelines. All experimental protocols were approved by the Institutional Animal Care and Use Committee at Tianjin International Joint Academy of Biomedicine accordance. A total of 1 × 10^7^ cells were injected subcutaneously to nude mice. When tumor volume reached approximately 50 mm^3^, mice were treated with 30 mg/kg of doxycycline and miR-17 mimics. The nude mice were monitored at three-day intervals for tumor appearance. Tumor size was calculated using the following equation: tumor size = width^2^ × length × 0.5. All mice were euthanized after four weeks of treatments.

### Immunohistochemical analysis

Tumor tissues from mice were fixed in 4% paraformaldehyde and embedded in paraffin. Thick slices were cut into 4 μm samples. Tissues were deparaffinized with xylene and dehydrated with ethanol of decreasing concentrations. Endogenous peroxidase activity was blocked with 3% hydrogen peroxide. Microwave antigen repair technique was utilized to retrieve antigens. After blocking, primary antibodies were incubated overnight at 4°C. After incubation for 1 h, tissue sections were washed with PBS and incubated with biotinylated goat anti-mouse IgG antibody (Zhongshan Biology Technology Co., Ltd., Beijing, China) at 37°C for 30 min. Following staining with 3,3′-diaminobenzidine/H_2_O_2_ and hematoxylin and eosin, sections were cleared and mounted for observation and analysis.

### Chromatin immunoprecipitation assay

A total of 1 × 10^7^ cells were plated in 15 cm culture dish. After treatment, cells were cross-linked using 1% formaldehyde (Sigma-Aldrich). Fixed cells were lysed, and chromatin was sheared using sonication. Chromatin fraction was incubated with NK-κB antibody overnight at 4°C. DNA was extracted and used in polymerase chain reaction (PCR) amplification with miR-17-specific primers.

### Luciferase activity assay

Cells were seeded in 96-well plates at a final concentration of 1 × 10^3^ cells per well and maintained at 37°C in 5% CO_2_, 24 h later, miRNA mimics were co-transfected in luciferase reporter plasmids containing miR-17 binding sites. 48 h after transfection, the medium was removed and the cells were lysed directly in the wells. Then 30μL cell lysate was added into the well of a white 96-well plate, followed by the addition of the firefly luciferase and the firefly luciferase activity was measured with a Dual-Glo^®^ luciferase assay system (Promega, Madison, WI, USA). Afterwards, stop reagent and the *Renilla* luciferase were added and the *Renilla* luciferase activity was determined by the same instrument. The relative luciferase activity was the ratio of the firefly luciferase activity to the *Renilla* luciferase activity. Mutations of promoter region of miR-17 were reference literature [39].

### Quantitative reverse transcription PCR (qRT-PCR) analysis

After treatment, cells were collected, and total RNAs were isolated using TRIzol (Sigma, St. Louis, MO, USA) per manufacturer's instructions. Approximately 2 μg RNAs were used in RT using miRNA-specific RT primers. cDNAs were then used to examine miR-17 expression. U6 small nuclear RNA was used as control. miR-17 expression levels were quantified using 2^−ΔΔCt^ methods. Each experiment was performed in triplicate.

### Western blotting

The cells were harvested, and proteins were extracted using radioimmunoprecipitation assay buffer (Beyotime, Jiangsu, China). Total proteins were separated using 10% sodium dodecyl sulfate polyacrylamide gel electrophoresis, transferred onto polyvinylidene difluoride membranes (Millipore, MA, USA) and blocked in blocking solution on a shaker for 2h at room temperature. After incubation with primary antibodies including PAR1(Affinity, dilution 1:1000), PI3K(Affinity, dilution 1:1000), AKT(Affinity, 1:1000), p-AKT(Affinity, 1:1000), NF-κB(Abcam, 1:2000), GAPDH(Affinity, 1:5000) and E-Cadherin(CST, 1:1000) diluted in blocking solution (5% nonfat milk dissolved in Tris-buffered saline (TBS)–Tween-20) overnight at 4°C, respectively. Membranes were incubated with horseradish-peroxidase-labeled secondary antibody (CST Inc., Danvers, MA, USA) (1:5000, diluted in blocking solution) for 1h at room temperature. Proteins were visualized using an enhanced chemiluminescence kit (Amersham Corp, Buckinghamshire, United Kingdom) and photographed.

### Statistical analysis

All results were presented as mean ± standard deviation. Values were analyzed using Student's t-test, ANOVA, and multivariate statistical analysis. Level of statistical significance was set at *P* < 0.05.

## References

[1] Mayor S (2015). Worldwide study links two new genetic variants to breast cancer. BMJ.

[2] Han W, Li W, Zhang X, Du Z, Liu X, Zhao X, Wen X, Wang G, Hu JF, Cui J (2017). Targeted breast cancer therapy by harnessing the inherent blood group antigen immune system. Oncotarget.

[3] Muss HB (2006). Targeted therapy for metastatic breast cancer. N Engl J Med.

[4] Heidari Z, Salouti M, Sariri R (2015). Breast cancer photothermal therapy based on gold nanorods targeted by covalently-coupled bombesin peptide. Nanotechnology.

[5] Sheppard VB, Cavalli LR, Dash C, Kanaan YM, Dilawari AA, Horton S, Makambi KH (2017). Correlates of Triple Negative Breast Cancer and Chemotherapy Patterns in Black and White Women With Breast Cancer. Clin Breast Cancer.

[6] Sokolosky ML, Stadelman KM, Chappell WH, Abrams SL, Martelli AM, Stivala F, Libra M, Nicoletti F, Drobot LB, Franklin RA, Steelman LS, McCubrey JA (2011). Involvement of Akt-1 and mTOR in sensitivity of breast cancer to targeted therapy. Oncotarget.

[7] Choi DH, Moon IS, Choi BK, Paik JW, Kim YS, Choi SH, Kim CK (2004). Effects of sub-antimicrobial dose doxycycline therapy on crevicular fluid MMP-8, and gingival tissue MMP-9, TIMP-1 and IL-6 levels in chronic periodontitis. J Periodontal Res.

[8] Zhong W, Chen S, Zhang Q, Xiao T, Qin Y, Gu J, Sun B, Liu Y, Jing X, Hu X, Zhang P, Zhou H, Sun T, Yang C (2017). Doxycycline directly targets PAR1 to suppress tumor progression. Oncotarget.

[9] Duivenvoorden WC, Popović SV, Lhoták S, Seidlitz E, Hirte HW, Tozer RG, Singh G (2002). Doxycycline decreases tumor burden in a bone metastasis model of human breast cancer. Cancer Res.

[10] Dhanesuan N, Sharp JA, Blick T, Price JT, Thompson EW (2002). Doxycycline-inducible expression of SPARC/Osteonectin/BM40 in MDA-MB-231 human breast cancer cells results in growth inhibition. Breast Cancer Res Treat.

[11] Qin Y, Zhang Q, Lee S, Zhong WL, Liu YR, Liu HJ, Zhao D, Chen S, Xiao T, Meng J, Jing XS, Wang J, Sun B (2015). Doxycycline reverses epithelial-to-mesenchymal transition and suppresses the proliferation and metastasis of lung cancer cells. Oncotarget.

[12] Yang E, Boire A, Agarwal A, Nguyen N, O'Callaghan K, Tu P, Kuliopulos A, Covic L (2009). Blockade of PAR1 signaling with cell-penetrating pepducins inhibits Akt survival pathways in breast cancer cells and suppresses tumor survival and metastasis. Cancer Res.

[13] Rahman A, True AL, Anwar KN, Ye RD, Voyno-Yasenetskaya TA, Malik AB (2002). Galpha(q) and Gbetagamma regulate PAR-1 signaling of thrombin-induced NF-kappaB activation and ICAM-1 transcription in endothelial cells. Circ Res.

[14] Bar-Shavit R, Maoz M, Kancharla A, Jaber M, Agranovich D, Grisaru-Granovsky S, Uziely B (2016). Protease-activated receptors (PARs) in cancer: novel biased signaling and targets for therapy. Methods Cell Biol.

[15] Han N, Jin K, He K, Cao J, Teng L (2011). Protease-activated receptors in cancer: A systematic review. Oncol Lett.

[16] Shah R (2009). Protease-activated receptors in cardiovascular health and diseases. Am Heart J.

[17] Croce CM, Calin GA (2005). miRNAs, cancer, and stem cell division. Cell.

[18] Hoppe R, Fan P, Büttner F, Winter S, Tyagi AK, Cunliffe H, Jordan VC, Brauch H (2016). Profiles of miRNAs matched to biology in aromatase inhibitor resistant breast cancer. Oncotarget.

[19] Yang S, Zhang H, Guo L, Zhao Y, Chen F (2014). Reconstructing the coding and non-coding RNA regulatory networks of miRNAs and mRNAs in breast cancer. Gene.

[20] Liao XH, Xiang Y, Yu CX, Li JP, Li H, Nie Q, Hu P, Zhou J, Zhang TC (2017). STAT3 is required for MiR-17-5p-mediated sensitization to chemotherapy-induced apoptosis in breast cancer cells. Oncotarget.

[21] Yu Z, Xu Z, Disante G, Wright J, Wang M, Li Y, Zhao Q, Ren T, Ju X, Gutman E, Wang G, Addya S, Li T (2014). miR-17/20 sensitization of breast cancer cells to chemotherapy-induced apoptosis requires Akt1. Oncotarget.

[22] Zhang ZJ, Ma SL (2012). miRNAs in breast cancer tumorigenesis (Review). Oncol Rep.

[23] Conev NV, Donev IS, Konsoulova-Kirova AA, Chervenkov TG, Kashlov JK, Ivanov KD (2015). Serum expression levels of miR-17, miR-21, and miR-92 as potential biomarkers for recurrence after adjuvant chemotherapy in colon cancer patients. Biosci Trends.

[24] Li H, Bian C, Liao L, Li J, Zhao RC (2011). miR-17-5p promotes human breast cancer cell migration and invasion through suppression of HBP1. Breast Cancer Res Treat.

[25] Chen C, Lu Z, Yang J, Hao W, Qin Y, Wang H, Xie C, Xie R (2016). MiR-17-5p promotes cancer cell proliferation and tumorigenesis in nasopharyngeal carcinoma by targeting p21. Cancer Med.

[26] Qu Y, Zhang H, Duan J, Liu R, Deng T, Bai M, Huang D, Li H, Ning T, Zhang L, Wang X, Ge S, Zhou L (2016). MiR-17-5p regulates cell proliferation and migration by targeting transforming growth factor-β receptor 2 in gastric cancer. Oncotarget.

[27] Khan S, Lopez-Dee Z, Kumar R, Ling J (2013). Activation of NFkB is a novel mechanism of pro-survival activity of glucocorticoids in breast cancer cells. Cancer Lett.

[28] Al-Halabi R, Bou Chedid M, Abou Merhi R, El-Hajj H, Zahr H, Schneider-Stock R, Bazarbachi A, Gali-Muhtasib H (2011). Gallotannin inhibits NFκB signaling and growth of human colon cancer xenografts. Cancer Biol Ther.

[29] Antoon JW, White MD, Slaughter EM, Driver JL, Khalili HS, Elliott S, Smith CD, Burow ME, Beckman BS (2011). Targeting NFκB mediated breast cancer chemoresistance through selective inhibition of sphingosine kinase-2. Cancer Biol Ther.

[30] Rayet B, Gélinas C (1999). Aberrant rel/nfkb genes and activity in human cancer. Oncogene.

[31] Lindskog C (2016). The Human Protein Atlas - an important resource for basic and clinical research. Expert Rev Proteomics.

[32] Pontén F, Jirström K, Uhlen M (2008). The Human Protein Atlas—a tool for pathology. J Pathol.

[33] Satelli A, Batth I, Brownlee Z, Mitra A, Zhou S, Noh H, Rojas CR, Li H, Meng QH, Li S (2017). EMT circulating tumor cells detected by cell-surface vimentin are associated with prostate cancer progression. Oncotarget.

[34] Saikali Z, Singh G (2003). Doxycycline and other tetracyclines in the treatment of bone metastasis. Anticancer Drugs.

[35] Su W, Li Z, Li F, Chen X, Wan Q, Liang D (2013). Doxycycline-mediated inhibition of corneal angiogenesis: an MMP-independent mechanism. Invest Ophthalmol Vis Sci.

[36] Agarwal A, Covic L, Sevigny LM, Kaneider NC, Lazarides K, Azabdaftari G, Sharifi S, Kuliopulos A (2008). Targeting a metalloprotease-PAR1 signaling system with cell-penetrating pepducins inhibits angiogenesis, ascites, and progression of ovarian cancer. Mol Cancer Ther.

[37] Alberti C, Pinciroli P, Valeri B, Ferri R, Ditto A, Umezawa K, Sensi M, Canevari S, Tomassetti A (2012). Ligand-dependent EGFR activation induces the co-expression of IL-6 and PAI-1 via the NFkB pathway in advanced-stage epithelial ovarian cancer. Oncogene.

[38] Carraro G, El-Hashash A, Guidolin D, Tiozzo C, Turcatel G, Young BM, De Langhe SP, Bellusci S, Shi W, Parnigotto PP, Warburton D (2009). miR-17 family of microRNAs controls FGF10-mediated embryonic lung epithelial branching morphogenesis through MAPK14 and STAT3 regulation of E-Cadherin distribution. Dev Biol.

[39] Zhou R, Hu G, Gong AY, Chen XM (2010). Binding of NF-kappaB p65 subunit to the promoter elements is involved in LPS-induced transactivation of miRNA genes in human biliary epithelial cells. Nucleic Acids Res.

